# Retraction: Cancer-associated fibroblasts-derived exosomes suppress immune cell function in breast cancer via the miR-92/PD-L1 pathway

**DOI:** 10.3389/fimmu.2024.1469689

**Published:** 2024-07-30

**Authors:** Frontiers Editorial Office

The journal retracts the 09 October 2020 article cited above.

Following publication, concerns were raised regarding the integrity of the images in the published figures. Images presented in this article were subsequently also identified in publications from unrelated author groups. The authors failed to provide a satisfactory explanation during the investigation, which was conducted in accordance with Frontiers’ policies. As a result, the data and conclusions of the article have been deemed unreliable and the article has been retracted.

Frontiers would like to thank Dr Sholto David for contacting the journal regarding the published article, the concerns regarding which were also documented on PubPeer.

This retraction was approved by the Chief Executive Editor of Frontiers. The authors received a communication regarding the retraction and agree to retract the article. This communication has been recorded by the publisher.

